# EGFR Signaling in Colorectal Cancer: Novel Therapeutic Strategies, Predictive Biomarkers, and Counteracting Treatment Resistance

**DOI:** 10.3390/ijms27073265

**Published:** 2026-04-03

**Authors:** Noura Abbas, Mohamad Mourad, Hiba Smaily, Layal Al Mahmasani, Ali Shamseddine

**Affiliations:** Department of Internal Medicine, Naef K. Basile Cancer Institute, American University of Beirut Medical Center, Riad El Solh, Beirut 1107-2020, Lebanon; na331@aub.edu.lb (N.A.); mm346@aub.edu.lb (M.M.); hs162@aub.edu.lb (H.S.); la108@aub.edu.lb (L.A.M.)

**Keywords:** EGFR, colorectal cancer, biomarkers, resistance, therapeutic strategies, cetuximab, panitumumab, liquid biopsy, precision oncology, review

## Abstract

Colorectal cancer (CRC) remains a leading cause of cancer morbidity and mortality worldwide, with nearly one quarter of patients presenting with metastatic disease at diagnosis. The epidermal growth factor receptor (EGFR) plays a central role in CRC pathogenesis through activation of downstream *RAS*/*RAF*/MAPK and PI3K/AKT/mTOR signaling pathways, and has become a major therapeutic target. Anti-EGFR monoclonal antibodies, cetuximab and panitumumab, have demonstrated survival benefit in selected patients, particularly those with left-sided, *RAS* wild-type tumors. However, primary and acquired resistance limit their efficacy, underscoring the need for predictive biomarkers and novel strategies. This review synthesizes current knowledge of EGFR biology, therapeutic integration, and biomarker development, including *RAS* and *BRAF* mutations, MSI status, *HER2* amplification, EGFR ligands (AREG/EREG), consensus molecular subtypes, and liquid biopsy applications. We also discuss mechanisms of resistance such as pathway reactivation, receptor mutations, and epithelial-to-mesenchymal transition, alongside emerging approaches, including combination regimens, ctDNA-guided rechallenge, and genotype-specific inhibitors. Collectively, these insights highlight the evolving landscape of precision oncology in CRC and the importance of molecular stratification to optimize EGFR-targeted therapy and overcome resistance.

## 1. Introduction

Colorectal cancer (CRC) ranks third in incidence with 1.9 million new cases, and ranks third in mortality during the year 2022, and around 24% of the cases present with de novo metastatic disease [1,2,3]. The epidermal growth factor receptor (EGFR) has a central role in promoting tumor growth, cell survival, and proliferation through downstream signaling pathways such as RAS/RAF/MEK/MAPK and the PI3K/AKT/mTOR pathways, and thus has emerged as a therapeutic target [4]. The CRYSTAL and PRIME trials are the first randomized trials to demonstrate the efficacy of the anti-EGFR antibodies cetuximab and panitumumab in the treatment of metastatic CRC (mCRC) and have led to improved survival outcomes [5,6]. However, the clinical benefit is limited to a subset of patients with mCRC: first-line use in right-sided colon cancers has been associated with poorer outcomes, and tumors harboring *RAS* mutations exhibit primary resistance. Clinical efficacy is primarily observed in patients with left-sided, *RAS* wild-type (WT) tumors, but responses are often short-lived due to the development of secondary (acquired) resistance [5,6,7].

Identifying predictive biomarkers of response or resistance is fundamental to optimizing the use of EGFR-targeted therapies. Since the presence of *RAS* mutations is the most well-established predictor of lack of response, their routine testing is essential prior to starting therapy [8,9,10]. Other biomarkers, such as *BRAF* mutations and other genomic or proteomic signatures, are under investigation to more precisely stratify patients and predict outcomes [11]. Consequently, strategies to overcome resistance are needed.

In this review, we will provide an overview of EGFR biology and signaling in CRC, highlighting the molecular mechanisms that drive tumor proliferation and survival, summarize the current landscape of anti-EGFR therapies, and discuss the predictive biomarkers and mechanisms of resistance, alongside emerging strategies aimed at overcoming resistance.

## 2. EGFR Biology and Signaling in CRC

### 2.1. The Biology

EGFR is a transmembrane glycoprotein involved in cellular proliferation, differentiation, and survival [12]. In 1960, it was discovered that the binding of EGF to its receptor significantly stimulates the proliferation of epidermal cells [13]. Aberrant EGFR signaling was strongly associated with the development of multiple cancers, including non-small cell lung cancer, CRC, pancreatic cancer, glioblastoma multiforme, and gastric cancer [14].

EGFR, commonly known as HER1 or ErbB1, belongs to the ErbB family of receptors, which also includes HER2 (ErbB2), HER3 (ErbB3), and HER4 (ErbB4) [15]. These receptors have a critical role in regulating cell proliferation and maintaining cellular functions through phosphorylation cascades and biochemical signaling [15]. Any dysregulation in these transmembrane protein receptors can lead to uncontrolled cell proliferation, tumorigenesis, and even metastasis [14].

In CRC, EGFR is frequently overexpressed, documented in 30–90% of advanced cases, and is associated with poor prognosis and inferior outcomes [12,16,17].

Seven EGFR ligands have been identified, including EGF, transforming growth factor-alpha (TGF-α), amphiregulin (AREG), epiregulin (EREG), betacellulin, heparin-binding EGF, and epigen [18]. Among these, AREG and EREG are particularly relevant in CRC biology and therapeutic response prediction [19].

### 2.2. EGFR Signaling Pathways in CRC

#### 2.2.1. RAS/RAF/MAPK/ERK Pathway

The RAS/RAF/MAPK/ERK pathway plays a crucial role in tumor cell survival and development, with ERK influencing tumor proliferation, invasion, metastasis, extracellular matrix degradation, and angiogenesis [20]. Ligand binding to the EGFR activates its intracellular tyrosine kinase, which then phosphorylates the receptor. The phosphorylated EGFR then binds GRB2, which also binds to SOS, leading to a complex formation [21,22]. This complex facilitates GDP–GTP exchange on RAS, activating RAF kinases (including BRAF). RAF then phosphorylates MEK, which in turn phosphorylates ERK, amplifying the signal and driving transcriptional programs that promote oncogenesis [23]. RAF, MEK and ERK are serine/threonine-selective protein kinases. They form a cascade that leads to a significant signal amplification. However, this cascade can be controlled via negative feedback.

Mutations in *KRAS* or *BRAF* disrupt negative feedback regulation, resulting in constitutive pathway activation and uncontrolled tumor growth [23]. While ERK activity is required for normal cellular development, its hyperactivation is a major contributor to cancer progression, underscoring the importance of this cascade in CRC biology.

#### 2.2.2. PI3K/AKT/PTEN/mTOR Pathway

The PI3K/AKT/mTOR pathway is another critical signaling axis in CRC, regulating protein synthesis, metabolism, and cell survival. Upon EGFR activation, Phosphatidylinositol 3-kinase (PI3K) phosphorylates membrane lipids to generate phosphatidylinositol-3,4,5-triphosphate (PIP-3), which recruits AKT to the plasma membrane. AKT is subsequently activated and phosphorylates downstream substrates, including mTOR, a key regulator of protein translation and cell cycle progression.

*PTEN*, a tumor suppressor, normally antagonizes PI3K activity by dephosphorylating PIP3. Loss-of-function mutations to *PTEN* or activating mutations in *PIK3CA*, present in 10–20% of CRC cases, result in unchecked pathway activation [24]. Clinically, phosphorylated AKT has been consistently linked to poor prognosis across multiple malignancies, including CRC, highlighting its relevance as both a biomarker and therapeutic target [25].

#### 2.2.3. Additional Pathways and Crosstalk

**Figure 1:**
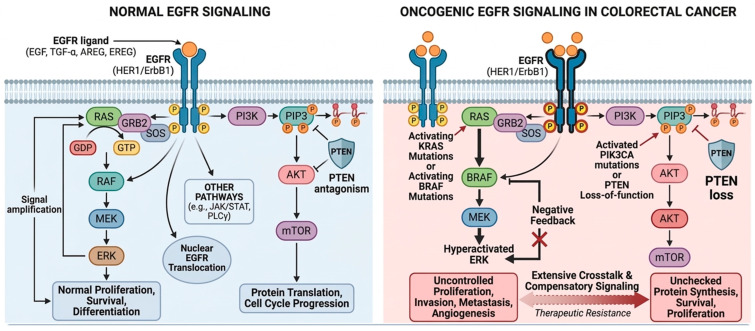
EGFR signaling pathways in CRC under normal and oncogenic conditions. AKT, Protein Kinase B; AREG, Amphiregulin; EGF, Epidermal Growth Factor; EGFR, Epidermal Growth Factor Receptor (HER1/ErbB1); ERK, Extracellular Signal-Regulated Kinase (MAPK); EREG, Epiregulin; GDP, Guanosine Diphosphate; GTP, Guanosine Triphosphate; GRB2, Growth factor Receptor-Bound Protein 2; JAK/STAT, Janus Kinase/Signal Transducer and Activator of Transcription; MEK, Mitogen-Activated Protein Kinase Kinase (MAP2K); mTOR, Mechanistic Target of Rapamycin; PI3K, Phosphoinositide 3-Kinase; PIP3, Phosphatidylinositol 3,4,5-trisphosphate; PLCγ, Phospholipase C-gamma; *PTEN*, Phosphatase and Tensin Homolog; *RAF*, Rapidly Accelerated Fibrosarcoma (serine/threonine kinase); *RAS*, Rat Sarcoma (small GTPase); SOS, Son of Sevenless (guanine nucleotide exchange factor); TGF-α, Transforming Growth Factor-alpha.

Beyond these two important cascades, EGFR signaling also engages the JAK/STAT pathway, particularly *STAT3* [26], which regulates transcription of genes involved in proliferation, angiogenesis, and immune evasion [27,28]. The phospholipase C-gamma (PLCγ) pathway contributes to calcium signaling and protein kinase C activation, while ligand-independent EGFR signaling can occur through nuclear translocation of the receptor, where it directly regulates gene expression related to DNA repair and cell cycle progression [29]. Extensive crosstalk between these pathways enables compensatory signaling when one cascade is inhibited, representing a major mechanism of therapeutic resistance [30] (Figure 1).

## 3. Anti-EGFR Therapeutic Landscape in CRC

### 3.1. Approved Monoclonal Antibodies

The monoclonal antibodies cetuximab and panitumumab represent the cornerstone of EGFR-targeted therapy in mCRC. Both bind with high affinity to the extracellular domain III of EGFR, preventing ligand binding, blocking receptor autophosphorylation, and promoting receptor internalization and degradation [31]. Clinical sensitivity to anti-EGFR antibodies depends not only on blocking receptor activation but also on the extent to which EGFR is internalized and degraded. Tumors that fail to achieve sufficient receptor depletion after antibody binding often exhibit primary resistance [32].

Structural differences in immunoglobulin isotypes confer distinct immunologic properties. Cetuximab is a chimeric mouse/human IgG1 antibody capable of inducing antibody-dependent cellular cytotoxicity (ADCC), adding an immune-mediated mechanism of tumor control. Panitumumab, in contrast, is a fully human IgG2 antibody, which reduces infusion-related reactions but lacks significant ADCC activity [31]. Both agents were initially approved based on efficacy in chemo-refractory disease. The pivotal BOND trial demonstrated improved response rates and survival with cetuximab [33], while early panitumumab studies confirmed activity in heavily pretreated patients [34].

### 3.2. Combination Strategies and Clinical Integration

The modern use of anti-EGFR therapy has shifted decisively toward combination regimens. Monotherapy is now rare, as combining anti-EGFR antibodies with chemotherapy doublets provides superior outcomes. In mCRC, the most common doublets are FOLFOX (fluorouracil/leucovorin plus oxaliplatin) or FOLFIRI (fluorouracil/leucovorin plus irinotecan). Both regimens exploit the cytotoxic effects of fluoropyrimidines on DNA synthesis, with oxaliplatin inducing DNA crosslinks and apoptosis, and irinotecan inhibiting topoisomerase I to prevent DNA replication and transcription. These regimens form the standard backbone of first-line therapy in mCRC and are frequently combined with anti-EGFR monoclonal antibodies. The CRYSTAL trial showed that cetuximab combined with FOLFIRI improved overall response rate (ORR) (ORR, 59.3% vs. 43.2%; odds ratio, 1.91; 95% confidence interval [CI], 1.24 to 2.93) and progression-free survival (PFS) (median PFS, 8.9 vs. 8.0 months; hazard ratio, 0.85; 95% CI, 0.72 to 0.99; *p* = 0.048) compared to FOLFIRI alone [5], while the PRIME trial demonstrated that panitumumab plus FOLFOX significantly prolonged PFS over FOLFOX alone (median PFS, 9.6 vs. 8.0 months; hazard ratio, 0.80; 95% CI, 0.66 to 0.97; *p* = 0.02) [35]. In first-line treatment of left-sided, *RAS* WT mCRC, these combinations achieve ORR of approximately 60% and a median PFS of 9–10 months [5,35].

Head-to-head comparisons with anti-VEGF therapy have further refined patient selection. The landmark PARADIGM trial (Japan, 2022) provided definitive evidence that in patients with *RAS* WT, left-sided mCRC, panitumumab plus mFOLFOX6 significantly improved overall survival (OS) compared with bevacizumab plus mFOLFOX6 (hazard ratio, 0.82; 95% CI 0.68–0.99; *p* = 0.031) [36]. Retrospective analyses of FIRE-3 and CALGB/SWOG 80405, reinforced by real-world studies, consistently show that left-sided *RAS* WT tumors derive substantial benefit from anti-EGFR therapy, with higher ORR (74% vs. 62%; odds ratio, 1.77; 95% CI, 1.39–2.26; *p* < 0.0001) and longer OS (hazard ratio, 0.77; 95% CI, 0.68–0.88; *p* < 0.0001) compared to bevacizumab [37]. In contrast, right-sided tumors, which often harbor *BRAF* mutations and CMS1 biology, respond poorly, even when there is *RAS* WT [38,39,40]. For these patients, anti-VEGF therapy remains the standard.

Molecular prerequisites are equally critical. Extended *RAS* testing (*KRAS* and *NRAS* exons 2–4) is mandatory, as activating mutations make EGFR blockade ineffective. *BRAF* V600E mutations, which are present in 8–12% of mCRC, confer poor prognosis and resistance to single-agent EGFR inhibition [41]. Combination strategies such as encorafenib plus cetuximab ± binimetinib (BEACON trial) and encorafenib plus cetuximab with mFOLFOX6(BREAKWATER trial) are reshaping treatment paradigms for this subgroup [42,43]. More recently, *KRAS* G12C inhibitors such as adagrasib and sotorasib, though modestly active as monotherapy in CRC, have shown enhanced efficacy when combined with anti-EGFR therapy, highlighting the expanding role of genotype-specific combinations [44].

Beyond the first line, anti-EGFR therapy continues to play a role in maintenance and rechallenge strategies. Trials such as VALENTINO and PANAMA show that after an initial course of FOLFOX plus panitumumab, stopping oxaliplatin and continuing with fluoropyrimidine (fluorouracil/leucovorin) plus panitimumab can help maintain disease control while reducing toxicity [45,46]. The concept of rechallenge has also become increasingly important. By using ctDNA tests to monitor when resistant *RAS* or *EGFR* mutations fade after stopping anti-EGFR therapy, clinicians can identify patients who may benefit from restarting treatment. Studies such as CRICKET and CAVE show that this approach can improve ORR and OS [47,48]. A summary of these trials is provided in Table 1.

## 4. Predictive Biomarkers of Response to Anti-EGFR Therapy

The predictive biomarkers for anti-EGFR therapy response and their respective implications are summarized in Table 2.

### 4.1. RAS and BRAF Mutations: Established Biomarkers

**Figure 2:**
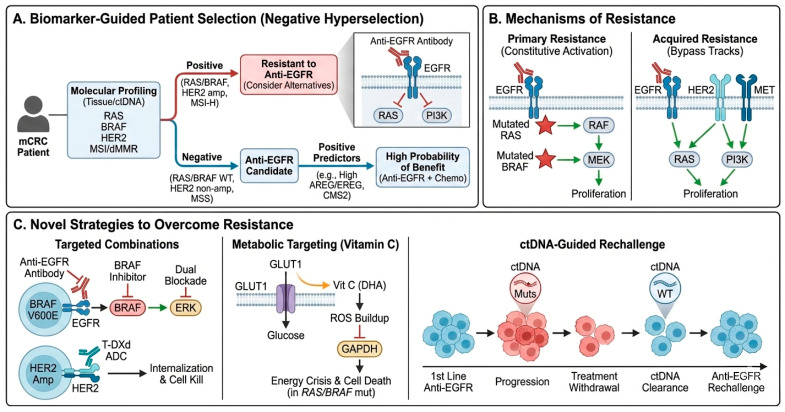
EGFR signaling in mCRC: Biomarkers, mechanisms of resistance, and novel strategies. ADC, Antibody Drug Conjugate; Amp, Amplification; AREG, Amphiregulin; *BRAF*, v-*RAF* Murine Sarcoma Viral Oncogene Homolog B1; CMS2, Consensus Molecular Subtype 2; ctDNA, Circulating Tumor DNA; dMMR, Deficient Mismatch Repair; DHA, Dehydroascorbic Acid (oxidized Vitamin C); EGFR, Epidermal Growth Factor Receptor (HER1/ErbB1); EREG, Epiregulin; Extracellular Signal-Regulated Kinase (MAPK); GAPDH, Glyceraldehyde-3-Phosphate Dehydrogenase; GLUT1, Glucose Transporter 1; HER2, Human Epidermal Growth Factor Receptor 2; MET, Hepatocyte Growth Factor Receptor; MEK, Mitogen-Activated Protein Kinase Kinase (MAP2K); mCRC, Metastatic Colorectal Cancer; MSI-H, Microsatellite Instability-High; MSS, Microsatellite Stable; Mut, Mutation; PI3K, Phosphoinositide 3-Kinase; *RAF*, Rapidly Accelerated Fibrosarcoma (serine/threonine kinase); *RAS*, Rat Sarcoma (small GTPase); ROS, Reactive Oxygen Species; T-DXd, Trastuzumab Deruxtecan (HER2-targeted antibody-drug conjugate); WT, Wild Type.

The discovery that *RAS* mutations predict resistance to anti-EGFR therapy represents a landmark achievement in precision oncology for CRC. Initially, only *KRAS* exon 2 (codons 12 and 13) mutations were recognized as negative predictive biomarkers [51]. However, expanded *RAS* testing to include *KRAS* exons 3 and 4 and *NRAS* exons 2, 3, and 4 revealed that approximately 15–20% of tumors classified as *KRAS* exon 2 WT harbor additional *RAS* mutations that similarly confer resistance [52]. Collectively, *RAS* mutations occur in ~50% of CRC cases, and these patients derive no benefit from anti-EGFR therapy due to constitutive activation of downstream MAPK signaling independent of EGFR status [53,54] (Figure 2).

*BRAF* V600E mutations, present in 8–12% of mCRC cases, are associated with poor prognosis and aggressive disease biology [41]. While initially considered a marker of anti-EGFR resistance similar to *RAS* mutations, the predictive value of *BRAF* V600E has proven more complex. Resistance is not absolute but requires targeted combination therapy (BRAF + MEK + EGFR inhibitors) to achieve clinical benefit, as demonstrated in the BEACON CRC (NCT02928224) [42] and BREAKWATER (NCT04607421) [43] trials. Other *BRAF* mutations (non-V600E, class II/III) may not confer the same degree of resistance and require individualized therapeutic consideration.

### 4.2. MSI-H/dMMR Status

Mismatch repair deficiency (dMMR) or microsatellite instability-high (MSI-H) tumors generally fail to respond to anti-EGFR therapy, even if *RAS* WT. Their high mutational burden and frequent subclonal resistance drivers make immune checkpoint inhibitors the preferred option, positioning MSI status as a strong negative predictive biomarker [55].

### 4.3. Negative Hyperselection and Extended Resistance Panels

While *RAS* and *BRAF* V600E testing effectively excludes about 50% of patients from anti-EGFR therapy, a substantial proportion of *RAS*/*BRAF* WT patients still fail to respond [56]. The concept of “negative hyperselection” has emerged to identify additional resistance mechanisms within the *RAS*/*BRAF* WT population. The PRESSING panel includes alterations in *HER2* (amplification or activating mutations), *MET* amplification, *NTRK*/*ROS1*/*RET* fusions, *PIK3CA* exon 20 mutations, *PTEN* inactivating alterations, *AKT1* mutations, EGFR ectodomain (EGFR-ECD) mutations, and *MAP2K1* mutations [57].

Studies implementing negative hyperselection demonstrate that 24–30% of *RAS*/*BRAF* WT patients harbor at least one additional resistance alteration [58,59]. Pietrantonio et al. [58] reported that in hyperselected patients without additional resistance alterations, ORR was 71% with a median PFS of 12.8 months, compared to 51% ORR and 7.6 months PFS in gene-altered patients. Similarly, Randon et al. [60] reported a median PFS of 12.8 months in hyperselected *RAS*/*BRAF* WT, microsatellite-stable patients without PRESSING2 alterations, compared with only 6.8 months in those harboring PRESSING2-defined resistance drivers, showing that these additional genomic alterations significantly worsen outcomes with anti-EGFR therapy. Comprehensive genomic profiling reveals that nearly half of patients with *RAS*/*BRAF* WT tumors harbor alternative resistance biomarkers when extensive panels are applied [61]. This underscores both the molecular heterogeneity of CRC and the potential value of comprehensive molecular profiling to optimize anti-EGFR therapy selection, though implementation challenges, including cost, tissue availability, and turnaround time, must be addressed.

### 4.4. HER2 Amplification

*HER2* amplification, present in 2–4% of *RAS* WT mCRC, provides an alternative survival signal and predicts resistance to anti-EGFR therapy [62]. Importantly, it is a positive predictor for HER2-targeted therapies such as trastuzumab deruxtecan, validated in DESTINY-CRC01 (NCT03384940) [63] and DESTINY-CRC02 (NCT04744831) [64].

### 4.5. EGFR Ligands: AREG and EREG as Predictive Biomarkers

AREG and EREG have emerged as promising predictive biomarkers for anti-EGFR therapy response. These EGFR ligands are frequently overexpressed in CRC, and their elevated expression correlates with improved outcomes following anti-EGFR treatment in *RAS* WT patients [65]. The biological rationale is that tumors with high AREG/EREG expression exhibit “EGFR addiction,” making them more dependent on EGFR signaling and consequently more sensitive to its inhibition [66].

Multiple retrospective and prospective studies have demonstrated that high mRNA expression of AREG and EREG in primary tumors associates with superior ORR, PFS, and OS in patients receiving anti-EGFR therapy [67,68]. Importantly, AREG/EREG status appears to provide independent predictive value beyond *RAS* mutation status, potentially helping identify the subset of *RAS* WT patients most likely to benefit.

Challenges remain in clinical implementation, including a lack of standardized assays, variability in cut-off values, and uncertainty regarding optimal sample source (primary tumor versus metastases). Most validation studies have been retrospective, and prospective trials specifically designed to test AREG/EREG-guided treatment selection are limited [19]. Ongoing efforts to develop robust, clinically validated assays may position AREG/EREG as valuable components of a multi-biomarker approach to optimize anti-EGFR therapy selection.

### 4.6. Consensus Molecular Subtypes (CMS) and Sidedness

Beyond single gene alterations, transcriptomic profiling has identified four CMS of CRC, each with distinct biology and therapeutic implications. CMS2 (canonical/epithelial) tumors, characterized by WNT and MYC pathway activation, are highly EGFR dependent and consistently derive substantial benefit from anti-EGFR therapy [69,70]. In contrast, CMS1 (immune) and CMS4 (mesenchymal) subtypes are associated with intrinsic resistance to EGFR blockade, with CMS1 tumors often harboring high mutational burden and immune infiltration, while CMS4 tumors exhibit stromal activation and epithelial-to-mesenchymal transition, both of which undermine EGFR dependence. CMS3 (metabolic) subtype may derive benefit from anti-EGFR therapy, but outcomes are less consistent than in CMS2.

### 4.7. Tumor Sidedness: Anatomical and Genetic Divergence

Although not a single molecular biomarker, primary tumor location (sidedness) has emerged as a powerful clinical predictor of response to anti-EGFR therapy [40]. Left-sided *RAS* WT tumors consistently derive substantial benefit, whereas right-sided tumors are largely resistant. This distinction reflects underlying biological differences rooted in embryology and molecular evolution.

The proximal colon (cecum, ascending colon, and proximal two-thirds of the transverse colon) originates from the midgut and is perfused by the superior mesenteric artery, while the distal colon (distal one-third of the transverse colon, descending colon, sigmoid colon, and rectum) derives from the hindgut and is perfused by the inferior mesenteric artery. This embryological divergence establishes a distinct vascular supply, lymphatic drainage, and epithelial biology, which contribute to different tumorigenic pathways [71].

Right-sided (proximal) colon cancers more frequently arise through the serrated neoplasia pathway, characterized by CpG island methylator phenotype (CIMP), epigenetic silencing of MLH1, and high microsatellite instability (MSI-H/dMMR). These tumors often harbor *BRAF* V600E mutations, *PIK3CA* alterations, CMS1 immune subtype biology, and exhibit hypermutated landscapes with high tumor mutational burden, correlating with poorer prognosis but enhanced susceptibility to immune checkpoint blockade. In contrast, left-sided tumors predominantly evolve through the chromosomal instability (CIN) pathway, following the classic adenoma-carcinoma sequence with *APC* loss, *KRAS* activation, and *TP53*/*SMAD4* inactivation. They are typically microsatellite-stable, enriched for EGFR ligand expression (AREG/EREG), display CMS2 canonical subtype biology, and are more often *RAS* WT, which explains their greater dependence on EGFR signaling and superior response to anti-EGFR therapy when *RAS*/*BRAF* WT [38,39,40].

Additionally, the right colon is exposed to higher concentrations of bile acids and enriched pro-inflammatory microbiota such as *Fusobacterium nucleatum*, which promote mutagenesis and immune evasion [71]. The left colon, by contrast, is more influenced by Wnt/β-catenin signaling and epithelial turnover dynamics.

**Figure 3:**
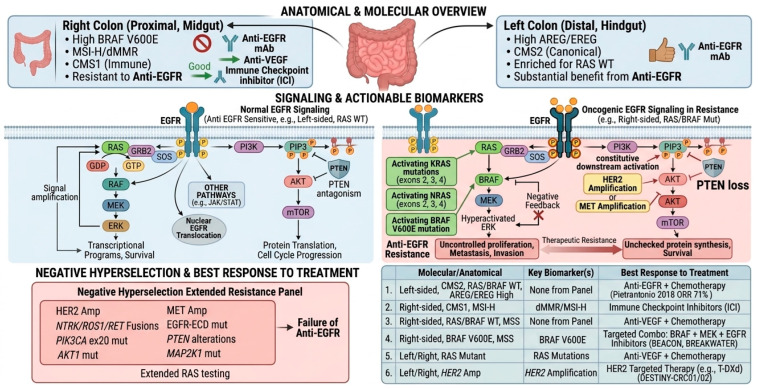
EGFR signaling in mCRC: Anatomical and molecular subtypes, biomarkers, and treatment strategies. *AKT*, Protein Kinase B; Amp, Amplification; AREG, Amphiregulin; *BRAF*, v-*RAF* Murine Sarcoma Viral Oncogene Homolog B1; CMS, Consensus Molecular Subtype; dMMR, Deficient Mismatch Repair; ECD, Extracellular Domain; EGF, Epidermal Growth Factor; EGFR, Epidermal Growth Factor Receptor (HER1/ErbB1); ERK, Extracellular Signal-Regulated Kinase (MAPK); EREG, Epiregulin; GDP, Guanosine Diphosphate; GRB2, Growth factor Receptor-Bound Protein 2; GTP, Guanosine Triphosphate; *HER2*, Human Epidermal Growth Factor Receptor 2; JAK/STAT, Janus Kinase/Signal Transducer and Activator of Transcription; *MET*, Hepatocyte Growth Factor Receptor; MEK, Mitogen-Activated Protein Kinase Kinase (MAP2K); mTOR, Mechanistic Target of Rapamycin; MSI-H, Microsatellite Instability-High; MSS, Microsatellite Stable; Mut, Mutation; *NTRK*, Neurotrophic Tyrosine Receptor Kinase; ORR, Objective Response Rate; P, Phosphorylation; PI3K, Phosphoinositide 3-Kinase; *PIK3CA*, Phosphatidylinositol-4,5-bisphosphate 3-Kinase Catalytic Subunit Alpha; PIP3, Phosphatidylinositol 3,4,5-trisphosphate; PLCγ, Phospholipase C-gamma; *PTEN*, Phosphatase and Tensin Homolog; *RAF*, Rapidly Accelerated Fibrosarcoma (serine/threonine kinase); *RAS*, Rat Sarcoma (small GTPase); *RET*, Rearranged during Transfection proto-oncogene; *ROS1*, c-ros Oncogene 1; SOS, Son of Sevenless (guanine nucleotide exchange factor); T-DXd, Trastuzumab Deruxtecan; TGF-α, Transforming Growth Factor-alpha; VEGF, Vascular Endothelial Growth Factor; WT, Wild Type.

Thus, sidedness is not merely anatomical but reflects deeper embryological, molecular, and microenvironmental heterogeneity that dictates prognosis and informs treatment selection. Left-sided tumors respond better to anti-EGFR therapy, while right-sided tumors often benefit more from anti-VEGF strategies or immunotherapy in the MSI-H/dMMR setting [38,39,40] (Figure 3, Table 3).

## 5. Liquid Biopsy and Circulating Tumor DNA (ctDNA) Applications

The introduction of liquid biopsy and ctDNA analysis has revolutionized molecular assessment in mCRC, addressing multiple limitations of tissue-based testing. Unlike static tissue assays, liquid biopsy offers minimally invasive sampling, captures tumor heterogeneity from multiple metastatic sites, enables real-time monitoring of molecular evolution, and allows serial assessments throughout therapy [72]. These features position ctDNA as both a detection method and a dynamic biomarker.

In the context of anti-EGFR therapy, ctDNA analysis has proven particularly valuable for three applications: (1) baseline selection for therapy initiation, (2) early assessment of treatment response, and (3) identification of candidates for rechallenge therapy [73,74].

### 5.1. Baseline Selection

Liquid biopsy and ctDNA can identify *RAS* and *BRAF* mutations with high concordance to tissue testing (≈90% agreement when ctDNA tumor fraction ≥ 1%). Importantly, ctDNA may detect resistance mutations present in metastatic sites but absent in the primary tumor, preventing futile anti-EGFR therapy [75,76]. The PARERE trial demonstrated that 34% of patients with tissue *RAS*/*BRAF* WT tumors harbored *RAS*/*BRAF* mutations in baseline ctDNA, and an additional 30% had other potential resistance alterations [77]. Therefore, ctDNA enhances patient selection by revealing hidden resistance drivers before therapy initiation.

### 5.2. Early Response Monitoring

Serial ctDNA monitoring provides dynamic information about treatment efficacy. The PLATFORM-B study showed that decreases in ctDNA trunk mutations at cycle 3 strongly predicted PFS (HR = 0.23, *p* = 0.001) [78]. Rising fractions of resistant *RAS*/*BRAF* mutations preceded clinical progression, allowing detection months before radiographic progression. Patients with “early molecular response” achieved a 77.5% response rate versus 25% in those with “early molecular progression” (*p* = 0.008). Thus, ctDNA serves as an early surrogate marker of treatment efficacy and progression risk.

### 5.3. Rechallenge Therapy

Resistance to anti-EGFR therapy often arises from acquired *RAS* or EGFR-ECD mutations, which fade after drug withdrawal. The CITRIC trial (EudraCT 2020-000443-31) [79] confirmed that ctDNA clearance of *RAS*, *BRAF*, and EGFR-ECD mutations predicts benefit from rechallenge. Patients with ctDNA-confirmed clearance had significantly improved PFS compared to those who remained ctDNA-positive, establishing ctDNA clearance as a validated biomarker for initiating third-line rechallenge. Similarly, in the RASINTRO study (NCT03259009) [80], anti-EGFR rechallenge in refractory disease was more effective in patients who were ctDNA *RAS*/*BRAF* WT at baseline, and especially in those showing an early molecular response (marked ctDNA decline > 50%). Together, these findings position ctDNA clearance as a predictive biomarker for both rechallenge eligibility and early treatment monitoring.

### 5.4. Emerging Applications

Emerging circulating biomarkers such as circulating non-coding RNAs (e.g., circ-EGFR) are also under investigation. These molecules act as microRNA sponges in the tumor microenvironment. Early studies suggest circ-EGFR can predict cetuximab response from a simple blood test. These emerging assays may expand the scope of liquid biopsy beyond DNA mutations to RNA-based predictive tools [81].

## 6. Mechanisms of Resistance

Despite the availability of targeted agents, such as cetuximab and panitumumab, resistance remains a major concern [56]. While these medications have improved survival in non-mutant *KRAS* WT mCRC, they are effective only in a subset of patients [56,82,83]. Resistance to these inhibitors is inevitable and typically develops within 1–2 years due to multiple molecular mechanisms [17,84].

### 6.1. Primary Resistance

Certain alterations in EGFR and EGFR-specific ligands that result in primary (inherent) resistance have been identified in recent years [85,86].

#### 6.1.1. *RAS* Family Mutations

*RAS* is a family of genes encoding GTP-binding proteins such as *KRAS*, *NRAS*, and *HRAS*. These proteins play significant roles in EGFR-related signaling pathways [87,88]. Mutations in the *RAS* genes can lead to downstream activation of effector pathways. These mutations are common in CRC, as they are positive in about 50% of CRC patients [89,90].

Exon 2-*KRAS* mutations:

Exon 2 *KRAS* mutations constitute 85–90% of all *KRAS* mutations [87]. Multiple trials have shown that patients with *KRAS* exon 2 mutations do not respond properly to anti-EGFR therapy [91,92].

Extended *RAS*: *NRAS* and other *KRAS* mutations:

In an updated analysis of the randomized phase III CRYSTAL study, researchers re-evaluated patients with *KRAS* exon 2 WT tumors to check for additional *RAS* mutations in *KRAS* exons 3 and 4, as well as *NRAS* exons 2, 3, and 4. Van Cutsem et al. discovered that approximately 15% of these patients actually harbored these extended mutations. The presence of these mutations predicted a poor response to the addition of cetuximab to chemotherapy, showing no significant improvement in PFS (7.2 vs. 6.9 months, HR = 0.81, *p* = 0.56) or OS (18.2 vs. 20.7 months, HR = 1.22, *p* = 0.50) [93].

#### 6.1.2. *RAF* Family Mutations

As mentioned above, a small proportion of patients with WT *RAS* mutations do not respond to anti-EGFR treatment. These patients often have mutations downstream in the EGFR signaling pathway. Among those are *BRAF* mutations, most commonly in the V600E allele [94]. This mutation leads to activation of the mitogen-activated protein kinase (MAPK) signaling pathway, which results in uncontrollable cell proliferation [95] (Figure 2).

#### 6.1.3. PIK3CA/PTEN Signaling Pathway Activation

Another downstream pathway of EGFR is the PIK3CA/PTEN signaling pathway. Mutations in *PIK3CA* and loss of *PTEN* expression often lead to activation of these pathways, eventually leading to tumor formation. This is often EGFR-independent and therefore does not respond to EGFR inhibition [96].

#### 6.1.4. JAK/STAT Signaling Pathway Activation

JAK and STAT are components of cytokine receptor signaling involved in cell proliferation, differentiation, and apoptosis [97]. *STAT3* is a transcription factor upregulated in some cancers, including CRC [97]. Continuous STAT3 activation driven by JAK-dependent cytokine signaling (both autocrine and paracrine) and tyrosine kinases like SRC and EGFR can drive cancer development. It significantly promotes tumor formation, blood vessel growth, tissue invasion, metastasis, and immune evasion [98].

#### 6.1.5. Epithelial-to-Mesenchymal Transition (EMT)

EMT is a complicated process where epithelial cells transform and acquire mesenchymal characteristics [99,100]. Buck et al. showed that CRC models maintaining a strict epithelial phenotype were 7-fold more sensitive to anti-EGFR treatment compared to those that had undergone EMT [101]. This can be explained by EGFR dependence; epithelial CRC cells maintain strong reliance on EGFR-RAS-MAPK signaling for proliferation and survival. Their intact epithelial phenotype, including E-cadherin–mediated adhesion, preserves receptor expression and downstream pathway activation, making them highly susceptible to EGFR blockade [101]. In contrast, during EMT, tumor cells undergo transcriptional reprogramming that reduces EGFR reliance and activates bypass pathways such as MET, TGF β, PI3K, and stromal interactions [102]. This shift diminishes EGFR dependence and confers resistance to anti-EGFR therapy. EMT can also emerge during therapy. Lin et al. [103] demonstrated that Smad4 loss promotes EMT and attenuates cetuximab sensitivity, while Bray et al. [104] observed increased EMT markers at the time of acquired cetuximab resistance in patient samples.

### 6.2. Acquired Resistance

Almost all patients with metastatic CRC initially respond to anti-EGFR treatment, but most tumors progress within 3–12 months [105]. The mechanisms of acquired resistance are the following:

Acquired mutations in the RAS/RAF signaling pathway:

In addition to their role in primary resistance, *KRAS* mutations constitute 50% of acquired resistance cases [106]. Misale et al. showed that out of 10 patients with *KRAS* WT metastatic CRC that progressed on cetuximab, 6 developed *KRAS* mutations [107].

Alternative growth factor receptor pathways activation:

Tumors were shown to evade EGFR inhibition by upregulation of alternative signaling pathways, and these include the following:

#### 6.2.1. IGF-1R Pathway Activation

IGF-1R is one of the receptors belonging to the transmembrane tyrosine kinase family. Upon its binding to its ligand, and activation of the *RAS*/*RAF*/MAPK and PI3K/AKT pathways takes place [108,109]. A strong association between the IGF-1R and EGFR pathway has been proven, as IGF-1R signaling has been shown to increase the activation of EGFR [110,111,112].

#### 6.2.2. *MET* Overexpression

The *MET* oncogene is the gene encoding the tyrosine kinase receptor for Hepatocyte Growth Factor (HGF). The activation of this receptor launches a cascade of intracellular signaling pathways, including the PI3K/AKT, RAC1/cell division control protein 42 (CDC42), driving cell proliferation and survival [113] (Figure 2).

#### 6.2.3. *HER2* Amplification

HER2 is a receptor tyrosine kinase that belongs to the same family as EGFR, the HER family. HER2 binding leads to activation of a signaling pathway common with that of EGFR, including the MAPK and PI3K/AKT pathways [114]. Bertotti et al. analyzed the genotype-response correlations in mCRC xenografts and found that *HER2* gene amplification was specifically related to cetuximab resistance, and that this resistance can be overcome by administration of a HER2 inhibitor [115] (Figure 2).

#### 6.2.4. *EGFR* S492R Mutation

The S492R mutation was first identified by Montagut et al. [116] as an acquired EGFR ectodomain mutation that emerges under selective pressure from cetuximab treatment. Mechanistically, the serine-to-arginine substitution at position 492 occurs within EGFR domain III, which is the binding site for cetuximab. This amino acid substitution alters the receptor conformation and disrupts the antibody–receptor interface, preventing cetuximab from binding effectively. As a result, cetuximab loses its inhibitory effect [117,118]. Importantly, panitumumab binds to a different epitope on EGFR and retains activity against tumors harboring the S492R mutation, which explains why patients with this mutation may still respond to panitumumab but not cetuximab [116,119]. This mechanism was further confirmed by structural studies, including Sickmier et al. [119], which demonstrated that the S492R mutation (also referred to as S468R depending on numbering convention) blocks cetuximab binding to EGFR domain III. Collectively, these findings establish *EGFR* S492R as a clinically relevant mechanism of acquired resistance to cetuximab.

#### 6.2.5. VEGF Signaling Pathway Alteration

Vascular endothelial growth factor is a signaling molecule driving angiogenesis. It binds to 3 different receptors: VEGFR1, VEGFR2, and VEGFR3. These receptors mediate changes in vascular proliferation and permeability [120]. Bianco et al. observed that cetuximab-resistant cells secreted higher levels of VEGF and VEGFR1 than the parental cetuximab-sensitive cells [121].

## 7. Strategies to Overcome Resistance

Several strategies have been developed to overcome both primary and acquired resistance to anti-EGFR therapy in CRC, focusing on combination therapies, molecularly guided rechallenge, and novel targeted approaches.

### 7.1. Rechallenge Strategies

Rechallenge with anti-EGFR antibodies is a simple yet effective method of treatment when combined with blood-based molecular testing. Recent evidence from a pooled analysis of four trials (CAVE, VELO, CRICKET, and CHRONOS) evaluated anti-EGFR rechallenge in 114 patients with refractory mCRC, with WT *RAS*/*BRAF* ctDNA. Rechallenge produced an overall response rate of 17.5% and a disease control rate of 72.3%, with a median PFS of 4.0 months and a median OS of 13.1 months. Notably, patients without liver metastases experienced significantly longer PFS and OS, suggesting the metastatic site may influence benefit [122]. In addition, a systematic review and meta-analysis evaluated anti-EGFR rechallenge in mCRC, including 13 clinical studies with 402 patients, mostly treated with cetuximab or panitumumab. The pooled analysis reported an objective response rate of 20.5%, a disease control rate of 67.4%, a median PFS of 3.5 months, and a median OS of 9.8 months. Patients with WT *RAS* ctDNA derived the greatest benefit, highlighting the role of ctDNA-guided patient selection [123]. Another systematic review of 14 studies, including 520 patients, evaluated anti-EGFR rechallenge in mCRC. The pooled analysis showed an objective response rate of 17.7% and a disease control rate of 61.7%, with median PFS of 2.4–4.9 months and median OS of 5–17.8 months [124].

### 7.2. KRAS Mutation

Strategies to incorporate KRAS inhibitors have been investigated to overcome resistance. Adagrasib, a *KRAS*-G12C inhibitor, was investigated in a phase 1/2 trial. Pretreated patients with *KRAS*-G12C-mutated mCRC received adagrasib monotherapy or adagrasib plus cetuximab. The objective response rate (ORR) was 19% and 46%, the median duration of response (DOR) was 4.3 months and 7.6 months, and the median PFS was 5.6 months and 6.9 months, respectively [125]. Similarly, sotorasib is another *KRAS*-G12C inhibitor that is investigated in mCRC. In the phase III CodeBreaK 300 trial, 160 pretreated patients with *KRAS* G12C–mutated mCRC were randomized to receive sotorasib 960 mg plus panitumumab, sotorasib 240 mg plus panitumumab, or investigator’s choice (trifluridine/tipiracil or regorafenib). After a median follow-up of 13.6 months, objective response rates were 30.2%, 7.5%, and 1.9%, respectively, and median OS was not reached for the 960 mg arm, 11.9 months for the 240 mg arm, and 10.3 months for the investigator’s choice [126].

High-dose intravenous (not oral) vitamin C can selectively induce apoptosis in *KRAS*- and *BRAF*-mutant CRC cells by exploiting their altered glucose metabolism. These mutant cells show increased GLUT1 expression and a strong reliance on aerobic glycolysis (Warburg effect) for energy and biosynthetic needs. The oxidized form of vitamin C, dehydroascorbate (DHA), enters cells through GLUT1/GLUT3 transporters and is reduced back to vitamin C, consuming glutathione, thioredoxin, and NADPH, which leads to ROS buildup, GAPDH inhibition, and an energy crisis that triggers cell death (Figure 2). Vitamin C also interferes with glycolysis by displacing *RAS* from the plasma membrane, blocking PKM2 phosphorylation, and reducing GLUT1 levels, further limiting glycolytic activity. This dual metabolic disruption may sensitize *KRAS*- and *BRAF*-mutant cells to treatment and help overcome resistance to anti-EGFR therapies [127]. A phase III randomized trial of previously untreated mCRC compared standard chemotherapy (FOLFOX ± bevacizumab) with or without high-dose intravenous vitamin C (1.5 g/kg/day for 3 days). In the whole cohort, the addition of vitamin C did not improve PFS, OS, or response rates. However, in prespecified subgroup analyses, patients with *RAS*-mutant tumors showed a significant PFS benefit when vitamin C was combined with chemotherapy (9.2 vs. 7.8 months; *p* = 0.01), suggesting a potential selective advantage in this molecular subset.

### 7.3. BRAF Mutation

In contrast to melanoma, the use of BRAF inhibitors alone has yielded modest outcomes in CRC [128]. However, the combination of BRAF inhibitors with other agents demonstrated improved outcomes. For instance, in the BEACON trial, patients were randomized to receive either a triplet with encorafenib, binimetinib, and cetuximab or a doublet with encorafenib and cetuximab or chemotherapy with cetuximab. The median OS was 9.0 months with triplet therapy versus 5.4 months in the control group, with a confirmed response rate of 26% compared with 2%. Doublet therapy with encorafenib and cetuximab also improved survival (median OS 8.4 months) relative to control [42]. In addition, the BREAKWATER trial compared encorafenib plus cetuximab combined with mFOLFOX6 demonstrated significant clinical benefit compared with standard care (chemotherapy with or without bevacizumab) versus standard care (chemotherapy with or without bevacizumab) in patients with untreated *BRAF*-mutated mCRC. Cetuximab + mFOLFOX6 significantly improved median PFS (12.8 vs. 7.1 months; *p* < 0.001) and OS (30.3 vs. 15.1 months *p* < 0.001) compared with standard care [43]. CodeBreak 301 is an ongoing phase III trial that is investigating sotorasib, panitumumab, and FOLFIRI versus FOLFIRI with or without bevacizumab in the first-line setting [129].

### 7.4. EGFR Mutation

EGFR mutation is a rare mutation in CRC but it can have a substantial impact on prognosis and treatment. At baseline, EGFR mutations in exons 18 to 21 occur at a frequency of 2.6%, 0.5%, 0.8%, and 3.8% [130]. However, the frequency of certain EGFR mutations can increase following treatment with anti-EGFR antibodies. For example, one study reported that the EGFR p.S492R mutation was absent at baseline but emerged at a rate of 1% after panitumumab therapy and 16% after cetuximab therapy [131]. EGFR mutations confer resistance to cetuximab, but panitumumab may remain effective depending on the specific mutation. For instance, mutations such as S492R, K467T, and R451C may respond to panitumumab, whereas G465E S464L, G465R, and I491M are associated with resistance [118,132].

The use of EGFR tyrosine kinase inhibitors (TKI) has been investigated in mCRC. In unselected patients, the use of erlotinib as maintenance treatment or the combination of lapatinib with capecitabine did not yield any significant results [133,134]. But in patients with EGFR mutations, there are case reports that confirm the efficacy of osimertinib with responses that may last more than one year [135,136].

### 7.5. PI3K/AKT Pathway, Including Activating PIK3CA Mutations and Loss of PTEN

Alterations in the PI3K/AKT pathway, including activating *PIK3CA* mutations and loss of *PTEN*, contribute to resistance to anti-EGFR therapy by activating survival signaling pathways parallel to EGFR-MAPK signaling. These aberrations may occur in *RAS* WT tumors and partially explain heterogeneous responses to cetuximab and panitumumab, with *PIK3CA* exon 20 mutations in particular being associated with reduced response and poorer outcomes [137]. Additionally, loss of *PTEN* expression, which releases negative regulation of the PI3K pathway and leads to constitutive AKT activation, has also been correlated with impaired responses to anti-EGFR therapy [138]. Early phase clinical trials evaluating PI3K or AKT inhibitors as a single agent or in combination, have demonstrated limited clinical activity and modest efficacy, largely restricted by toxicity. These agents have not shown sufficient benefit to progress to successful phase III development in unselected CRC populations, underscoring the need for improved biomarker-driven patient selection and rational combination strategies [139].

### 7.6. Human Epidermal Growth Factor Receptor 2 (HER-2) Amplification

*HER2* amplification functions as a bypass mechanism of resistance to EGFR inhibitors by activating parallel ERBB family signaling pathways independent of EGFR, sustaining downstream MAPK and PI3K/AKT signaling that renders cetuximab and panitumumab less effective in mCRC even in patients with *RAS*/*BRAF* WT tumors [62]. *HER2* amplification is present in approximately 2–4% mCRC [62]. Several studies have investigated HER-2 blockade in mCRC. In the MyPathway basket study, patients with treatment-refractory, *HER2*-amplified mCRC received dual HER2 blockade with pertuzumab and trastuzumab. Among 57 evaluable patients, the primary endpoint was ORR and was 32%, including one complete response and 17 partial responses [140]. Another study (phase II) randomized patients with *RAS*/*BRAF* WT, HER2-positive mCRC to receive either trastuzumab plus pertuzumab or cetuximab plus irinotecan, with crossover to dual HER2 blockade permitted after progression. Although primary endpoint which was the median PFS was similar between treatment arms (4.7 vs. 3.7 months, respectively), dual HER2 inhibition achieved a higher ORR of 34.6% compared to 28% respectively, noting that the patients who responded to treatment had significantly higher HER2/CEP17 ratio (14.7 vs. 6.5; *p* = 0.005) and gene copy number (29.7 vs. 13.2, *p* = 0.004) compared to non-responders [141]. The HERACLES-A evaluated dual HER2 blockade with trastuzumab and lapatinib in patients with *KRAS* WT, chemo-refractory HER2-positive mCRC. Among 32 evaluable patients, the objective response rate was 28%, including one complete response and eight partial responses, with an additional 41% achieving stable disease. Median PFS was 4.7 months and median OS was 10.0 months, with one patient maintaining a complete response beyond 7 years of follow-up [142]. HERACLES-B was another single-arm phase II trial evaluating dual HER2-targeted therapy with pertuzumab and trastuzumab-emtansine (T-DM1) in patients with *RAS*/*BRAF* WT, HER2-positive mCRC refractory to standard treatments. Thirty-one heavily pretreated patients received pertuzumab plus T-DM1 until progression or toxicity, with objective response rate as the primary endpoint. The trial did not meet its prespecified ORR threshold, achieving an ORR of 9.7%, although a high disease control rate (67.7%) and a median PFS of 4.1 months were observed. To note that clinical benefit (response or stable disease) lasting more than 4 months, was significant in tumors with high *HER2* expression 3+ compared with those showing lower expression (2+) (*p* = 0.03) [143]. DESTINY-CRC02 was a multicenter, randomized phase II study evaluating two doses of the antibody–drug conjugate trastuzumab deruxtecan (5.4 mg/kg vs. 6.4 mg/kg) in patients with heavily pretreated, HER2-positive mCRC, including both *RAS* WT and *RAS*-mutant tumors, with the ORR as the primary endpoint. Trastuzumab deruxtecan demonstrated antitumor activity, with an ORR of 37.8% in the 5.4 mg/kg dose compared to 27.6%. Subgroup analysis did not show any difference between patients who are *KRAS* mutated and *KRAS* WT [64]. In addition, HER-2 testing can be done using ctDNA. One phase II study evaluated dual HER2 blockade with pertuzumab and trastuzumab in patients with mCRC selected for *HER2* amplification using either tumor tissue or circulating tumor DNA genotyping. The trial met its primary endpoint, demonstrating comparable objective response rates in tissue-positive (30%) and ctDNA-positive (28%) patients, while no responses were observed in a matched real-world control cohort treated with standard salvage therapy [144].

### 7.7. MET Amplification and HGF Overexpression

Amplification of *MET* or overexpression of its ligand HGF leads to activation of MAPK and PI3K pathways despite EGFR inhibition, promoting tumor growth and survival [145,146]. This mechanism is predominantly associated with acquired resistance following initial response to anti-EGFR therapy [145]. Preclinical data support combined inhibition of EGFR and MET as a strategy to overcome this resistance pathway [146].

### 7.8. Tumor Heterogeneity and Clonal Evolution

In patients with mCRC who are receiving anti-EGFR therapy, circulating tumor DNA profiling reveals the proliferation of resistant subclones harboring mutations in *KRAS*, *NRAS*, *MET*, *ERBB2*, *FLT3*, *EGFR*, and *MAP2K1*. These alterations often regress following withdrawal of EGFR inhibition, highlighting the dynamic nature of clonal evolution underlying adaptive resistance. Reintroduction of anti-EGFR monoclonal antibodies can lead to renewed treatment responsiveness, supporting the use of ctDNA-based approaches to guide EGFR rechallenge [147] (Figure 2).

## 8. Future Directions

Future advances in overcoming resistance to anti-EGFR therapy in mCRC will increasingly rely on refined molecular stratification, dynamic biomarker monitoring, and rational combination strategies. Whereas conventional *RAS* and *BRAF* testing remains fundamental for initial patient selection, expanding comprehensive genomic profiling to include alterations such as *HER2* amplification, *MET* amplification, and other genetic variations enables a strategy of patient hyperselection. Patients selected through this extended molecular approach will achieve higher response rates and improved clinical outcomes [148]. Furthermore, real-time monitoring through liquid biopsies will transform management paradigms by detecting the emergence of acquired resistance mutations prior to clinical progression. This will permit personalized treatment strategies, including treatment discontinuation, therapeutic switching, or rechallenge with anti-EGFR agents once resistant clones decay [148].

Innovative therapeutic modalities targeting both the EGFR axis and intersecting pathways are under active investigation. Next-generation EGFR-targeted agents offer potential to overcome canonical resistance mechanisms. Oligoclonal antibodies such as MM-151 can bind to the EGFR extracellular domain at multiple regions. This antibody has shown a response in patients who developed resistance after treatment with anti-EGFR antibodies [149]. Bispecific antibodies such as Amivantamab, which targets EGFR-MET, have demonstrated efficacy when combined with chemotherapy in right-sided CRC [150]. Furthermore, several anti-EGFR antibody drug conjugates are under investigation for the treatment of CRC [151]. Additionally, given that *KRAS* represents the predominant oncogenic driver in pancreatic cancer and a key mediator of resistance to anti-EGFR therapies, novel therapeutic approaches such as pan-KRAS inhibitors are currently under active investigation. These agents have shown encouraging preliminary activity and may help restore sensitivity to EGFR inhibition, particularly when used in combination with anti-EGFR therapies [152].

## 9. Conclusions

EGFR-targeted therapy has transformed the management of mCRC, but its benefit is limited to carefully selected patients. *RAS* and *BRAF* mutations, MSI status, and tumor sidedness remain the strongest predictors of resistance, while newer biomarkers such as PRESSING panels, CMS classification, and EGFR ligands are refining patient selection. The rise in liquid biopsy and ctDNA monitoring is a major advance, allowing real-time tracking of tumor evolution, early detection of resistance, and implementation of rechallenge strategies. Moving forward, combining ctDNA dynamics with transcriptomic profiling and genotype-specific therapies offers the best chance to overcome resistance and extend the durability of EGFR blockade. The future of EGFR therapy lies not in broader application but in deeper precision, using smarter biomarker strategies to maximize benefit, avoid ineffective treatment, and improve both survival and quality of life for patients.

## Figures and Tables

**Table 1:** Major anti-EGFR trials in mCRC.

Trial (NCT)	Phase	Intervention(s)	Population	Main Findings
BOND, 2004 [33]	Phase II	Cetuximab ± irinotecan	Chemo-refractory mCRC	Cetuximab improved response rates and survival in irinotecan-refractory patients.
CRYSTAL (NCT00154102), 2009 [5]	Phase III	Cetuximab + FOLFIRI vs. FOLFIRI	First-line mCRC, *KRAS* WT	The addition of cetuximab improved ORR and PFS.
PRIME (NCT00364013), 2010 [35]	Phase III	Panitumumab + FOLFOX vs. FOLFOX	First-line mCRC, *KRAS* WT	Panitumumab improved PFS; benefit restricted to *KRAS* WT.
PARADIGM (NCT02394795), 2022 [36]	Phase III	Panitumumab + mFOLFOX6 vs. bevacizumab + mFOLFOX6	First-line, *RAS* WT, left-sided mCRC	Panitumumab significantly improved OS over bevacizumab.
FIRE-3 (NCT00433927), 2014 [49]	Phase III	Cetuximab + FOLFIRI vs. bevacizumab + FOLFIRI	First-line mCRC, *KRAS* WT	Cetuximab improved OS in *KRAS* WT, especially in left-sided tumors.
CALGB/SWOG 80,405 (NCT00265850), 2017 [50]	Phase III	Cetuximab vs. bevacizumab + chemo (FOLFOX/FOLFIRI)	First-line mCRC, *KRAS* WT	No OS difference overall; sidedness analysis showed benefit for left-sided tumors with cetuximab.
BEACON (NCT02928224), 2019 [42]	Phase III	Encorafenib + cetuximab ± binimetinib vs. irinotecan or FOLFIRI + cetuximab	*BRAF* V600E mCRC	Triplet improved OS and ORR compared to control.
BREAKWATER (NCT04607421), 2025 [43]	Phase III	Encorafenib + cetuximab + mFOLFOX6	First-line *BRAF* V600E mCRC	Early data suggest improved outcomes; definitive results pending.
VALENTINO (NCT02476045), 2019 [45]	Phase II	Panitumumab + FOLFOX induction → maintenance	*RAS* WT mCRC	Maintenance with panitumumab + fluoropyrimidine (fluorouracil/leucovorin) maintained disease control and reduced toxicity.
PANAMA (NCT01991873), 2022 [46]	Phase II	Panitumumab + FOLFOX induction → maintenance	*RAS* WT mCRC	Supported maintenance strategy with panitumumab + fluoropyrimidine (fluorouracil/leucovorin).
CRICKET (NCT02296203), 2019 [48]	Phase II	Cetuximab rechallenge	*RAS* WT mCRC, prior EGFR exposure	ctDNA-guided rechallenge yielded ORR ~21%, benefit in patients with *RAS* WT ctDNA.
CAVE (NCT04561336), 2021 [47]	Phase II	Cetuximab + avelumab (PD-L1 inhibitor)	*RAS* WT mCRC, post-EGFR therapy	Rechallenge with cetuximab + immunotherapy showed a promising OS benefit.

ctDNA, Circulating Tumor DNA; FOLFIRI, Folinic acid (leucovorin), Fluorouracil (5-FU), Irinotecan; FOLFOX, Folinic acid (leucovorin), Fluorouracil (5-FU), Oxaliplatin; mCRC, Metastatic Colorectal Cancer; mFOLFOX6, Modified FOLFOX6 regimen; ORR, Objective Response Rate; OS, Overall Survival; PD-L1, Programmed Death-Ligand 1; PFS, Progression-Free Survival; WT, Wild Type.

**Table 2:** Predictive biomarkers of response to anti-EGFR therapy in mCRC.

Biomarker Target	Testing Modality	Predictive Role	Clinical Implication
* RAS * (*KRAS*/*NRAS*) Mutations	Tissue NGS/ctDNA	Strong Negative	Absolute contraindication for anti-EGFR therapy.
* BRAF * V600E Mutation	Tissue NGS/ctDNA	Negative (Monotherapy)	Directs treatment toward BRAF + EGFR inhibitor combinations.
* HER2 * Amplification	Tissue NGS/IHC/FISH	Strong Negative	Directs treatment toward HER2-targeted ADCs or combinations.
dMMR/MSI-H	IHC/PCR/NGS	Strong Negative	Diverts to Immune Checkpoint Inhibitor (ICI) therapy.
AREG/EREG Expression	IHC/mRNA	Strong Positive	Indicates high EGFR-dependence; predicts superior survival benefit.
ctDNA Mutational Clearance	Liquid Biopsy (ctDNA)	Strong Positive	Gateway biomarker for confirming eligibility for anti-EGFR rechallenge.

AREG, Amphiregulin (EGFR ligand); ctDNA, Circulating Tumor DNA; dMMR, Deficient Mismatch Repair; EGFR, Epidermal Growth Factor Receptor; EREG, Epiregulin (EGFR ligand); FISH, Fluorescence In Situ Hybridization; *HER2*, Human Epidermal Growth Factor Receptor 2; IHC, Immunohistochemistry; MSI-H, Microsatellite Instability-High; NGS, Next-Generation Sequencing; PCR, Polymerase Chain Reaction.

**Table 3:** Genetic and biological differences between left- and right-sided CRC.

Feature	Left-Sided CRC (Distal)	Right-Sided CRC (Proximal)
Embryological origin	Hindgut; supplied by the inferior mesenteric artery	Midgut; supplied by the superior mesenteric artery
Dominant pathway	Chromosomal Instability (CIN)—adenoma-carcinoma sequence	Serrated neoplasia pathway—CpG island methylator phenotype (CIMP)
Key driver mutations	*APC* loss → *KRAS* activation → TP53/SMAD4 loss	*MLH1* promoter hypermethylation → MSI-H/dMMR; frequent *BRAF* V600E, *PIK3CA*, *TGFBR2* mutations
Genomic profile	Microsatellite stable (MSS); lower tumor mutational burden	MSI-H/dMMR; hypermutated (TMB-high)
Consensus Molecular Subtypes (CMS)	CMS2 (canonical/epithelial); EGFR-dependent	CMS1 (immune) and CMS3 (metabolic); immune-driven biology
EGFR ligand expression (AREG/EREG)	High; predicts sensitivity to anti-EGFR therapy	Low; reduced EGFR dependence
Microenvironmental influences	Wnt/β-catenin signaling; epithelial turnover	Higher bile acid exposure; enriched Fusobacterium nucleatum, promoting inflammation and immune evasion
Therapeutic implications	Better response to anti-EGFR therapy (if *RAS*/*BRAF* WT)	Poor response to EGFR blockade; benefit from anti-VEGF or immune checkpoint inhibitors (MSI-H/dMMR)
Prognosis	Generally more favorable	Often worse, especially in metastatic disease

*APC*, Adenomatous Polyposis Coli; AREG, Amphiregulin; *BRAF*, v-*RAF* Murine Sarcoma Viral Oncogene Homolog B1; CRC, Colorectal Cancer; EGFR, Epidermal Growth Factor Receptor; EREG, Epiregulin; *MLH1*, MutL homolog 1; MSI-H/dMMR, Microsatellite Instability-High/Deficient Mismatch Repair; *PIK3CA*, Phosphatidylinositol-4,5-bisphosphate 3-Kinase Catalytic Subunit Alpha; *SMAD4*, Mothers Against Decapentaplegic Homolog 4; *TGFBR2*, Transforming Growth Factor Beta Receptor 2; *TP53*, Tumor Protein p53; TMB, Tumor Mutational Burden; VEGF, Vascular Endothelial Growth Factor; WT, Wild Type.

## Data Availability

No new data were created or analyzed in this study. Data sharing is not applicable to this article.
